# The transferability of laparoscopic and open surgical skills to robotic surgery

**DOI:** 10.1186/s41077-022-00223-2

**Published:** 2022-09-05

**Authors:** Maria Ordell Sundelin, Charlotte Paltved, Pernille Skjold Kingo, Henrik Kjölhede, Jørgen Bjerggaard Jensen

**Affiliations:** 1 Department of Clinical Medicine, Health, Aarhus University, Aarhus, Denmark; 2 Corporate MidtSim, Central Denmark Region, Aarhus, Denmark; 3 Department of Urology, Aarhus University Hospital, Aarhus, Denmark; 4 Department of Urology, Sahlgrenska University Hospital, Göteborg, Sweden; 5 Department of Urology, Institute of Clinical Sciences, Sahlgrenska Academy, University of Gothenburg, Göteborg, Sweden

**Keywords:** Simulation training, Surgical training, Laparoscopy, Open surgery, Minimally invasive surgery, Robotics, Skill transfer, Assessment

## Abstract

**Background:**

Within the last decades, robotic surgery has gained popularity. Most robotic surgeons have changed their main surgical activity from open or laparoscopic without prior formal robotic training. With the current practice, it is of great interest to know whether there is a transfer of surgical skills. In visualization, motion scaling, and freedom of motion, robotic surgery resembles open surgery far more than laparoscopic surgery. Therefore, our hypothesis is that open-trained surgeons have more transfer of surgical skills to robotic surgery, compared to surgeons trained in laparoscopy.

**Methods:**

Thirty-six surgically inexperienced medical students were randomized into three groups for intensive simulation training in an assigned modality: open surgery, laparoscopy, or robot-assisted laparoscopy. The training period was, for all study subjects, followed by performing a robot-assisted bowel anastomosis in a pig model. As surrogate markers of surgical quality, the anastomoses were tested for resistance to pressure, and video recordings of the procedure were evaluated by two blinded expert robotic surgeons, using a global rating scale of robotic operative performance (Global Evaluative Assessment of Robotic Skills (GEARS)).

**Results:**

The mean leak pressure of bowel anastomosis was 36.25 (7.62–64.89) mmHg in the laparoscopic training group and 69.01 (28.02–109.99) mmHg in the open surgery group, and the mean leak pressure for the robotic training group was 108.45 (74.96–141.94) mmHg. The same pattern was found with GEARS as surrogate markers of surgical quality. GEARS score was 15.71 (12.37–19.04) in the laparoscopic training group, 18.14 (14.70–21.58) in the open surgery group, and 22.04 (19.29–24.79) in the robotic training group. In comparison with the laparoscopic training group, the robotic training group had a statistically higher leak pressure (*p* = 0.0015) and GEARS score (*p* = 0.0023). No significant difference, for neither leak pressure nor GEARS, between the open and the robotic training group.

**Conclusion:**

In our study, training in open surgery was superior to training in laparoscopy when transitioning to robotic surgery in a simulation setting performed by surgically naive study subjects.

## Introduction

Robotic surgery has represented a paradigm shift for surgical practice towards minimally invasive procedures. With the exception of a few young surgeons having completed a formal robotic training program during their residency, most European surgeons have changed their main surgical activity from open or laparoscopic to robotic surgery without prior formal robotic training.

With the limited time available for training and the current practice, the extent and whether there is a transfer of skills from other surgical modalities become highly topical. The translational aspect from other surgical modalities to robotic surgery remains ambiguous.

Studies addressing the question of transferability have produced conflicting results.

A study by Kowalewski et al. that compared laparoscopic and open surgery experience on robotic surgery skills found that robotic-assisted surgery requires skills distinct from both conventional laparoscopy and open surgery [1]. However, some studies have found a transfer of skills from laparoscopy to robotic surgery [2, 3], as demonstrated by the findings that skilled laparoscopic surgeons were able to achieve comparable initial results to those of surgeons who have performed more robotic-assisted procedures. Other research has shown the transfer of skills from open surgery to robotic surgery [4–6]. Similar conclusions were reached by Cumpanas et al. [7] who found that surgeons experienced in open surgical techniques can directly proceed into robotic surgery as this modality imitate many of the tools and techniques required in open surgery. Therefore, the question of whether past open and laparoscopic experience influences the surgeons’ learning curves for robotic surgery is still discussed.

Guidelines have been developed by surgical associations about training surgeons for practicing robotic surgery [8–10], but it is still the individual institutions’ responsibility credentialing surgeons for practicing robotic surgery. Laparoscopy is still widely used as part of many training curricula worldwide for proficiency in robot surgery, despite the limited evidence on the transferability of conventional laparoscopic skills.

In the nature of robotic-assisted surgery lies a three-dimensional surgical view allowing depth perception, motion scaling, wristed instrumentation, and seven degrees of freedom of motion, which resembles open procedures far more than laparoscopic surgery [11].

Our hypothesis in the present study was that open-trained surgeons have better conditions to transfer the surgical skills to robotic surgery, compared to surgeons trained in laparoscopy.

## Materials and methods

Through an active application process, 36 medical students from Aarhus University were included in the study. Study subjects had no prior surgical experience and little or no experience with surgical simulation training. Study participants were randomly assigned to one of three groups: laparoscopic, open surgery, or robotic training. The training setup has previously been described [12]. In summary, they were given a baseline evaluation (pre-training test) in the surgical modality to which they were assigned. The laparoscopy group did a peg transfer task (Fundamentals of Laparoscopic Surgery Program, Los Angeles, CA). The open surgery group did a low-fidelity simulated exercise with knot tying, and the robotic group did a virtual task on the dVSS-Trainer (The da Vinci Skills Simulator).

Afterwards, they participated in a 6-week training regimen comprising a 90-min repeated procedure per week. In the assigned surgical modality, study participants received supervised and standardized instruction in suturing small bowel anastomosis. Fresh pig small intestine fragments (10 cm) were harvested and kept frozen. A number of segments were thawed at the beginning of every session. A seromuscular stitching approach with a running 4-0 monofilament thread was used to complete an end-to-end anastomosis aligning bisected segments. This model has previously been reported as a suitable bowel anastomosis surgical simulation model [13].

At the end of the training period, the study subjects did a post-training test in the surgical modality they were trained, using the same test as at the baseline assessment (pre-training test).

**Fig. 1:**
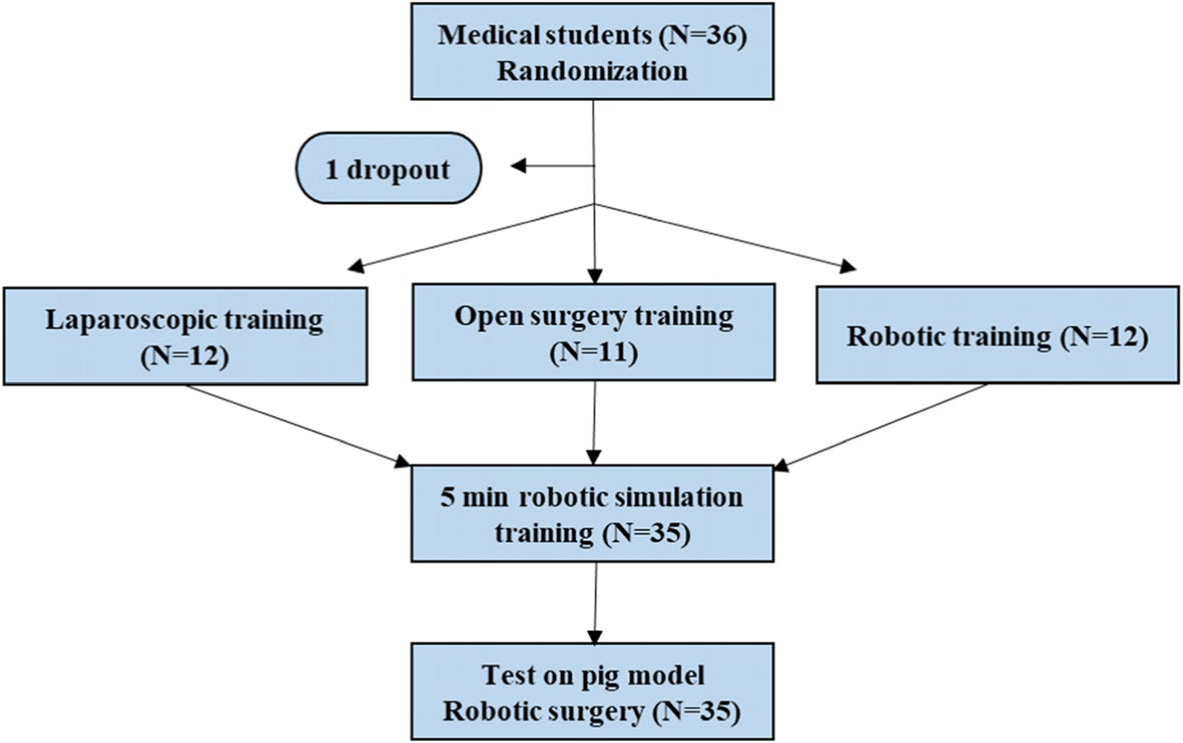
Study flow diagram

As a test for the study purpose of investigation of surgical skills in a robotic procedure, study participants were evaluated in required skills on an anesthetized pig (Fig. 1). The robotic-trained group served as a control group. All participants had to complete a robotic-assisted small bowel end-to-end anastomosis within a 90-min time constraint. Prior to the evaluation, all study participants completed a 5-min basic handling training session in the robotic dVSS trainer to replicate novice robotic skills. The intestines were harvested, and leak pressure was tested by occluding the one end of the bowel 3 cm from the anastomosis. The opposite end of the intestinal lumen was connected to a tube and bag filled with a 1000-ml water. Raising the water bag progressively increased the pressure. When a suture line leak was discovered, the height of the bag was recorded and the pressure calculated using the following formula: height × water density × Earth’s gravity = pressure. Anastomoses that were obviously leaky were registered with a leak pressure of 0. Two professional robotic surgeons blinded to the study participants’ training group and identity reviewed video recordings of the final study test performed by the study subjects, using a validated assessment tool for rating technical skills in robotic surgery, GEARS [14]. This 30-point global rating scale consists of six components: depth perception, bimanual skill, efficiency, force control, autonomy, and robot control. Each was scored 1 to 5 on an anchored Likert scale, where higher scores indicate higher proficiency.

The study was carried out at Aarhus University’s Department of Clinical Medicine. Anesthesia, analgesia, and euthanasia were carried out as described in the study by Kingo et al. [15].

### Statistical analysis

If not stated otherwise, parametric data were evaluated as means with 95% confidence intervals. The generalized linear model ANOVA was used to compare the groups, with leak pressure and GEARS score as dependent factors and surgical modality as independent variables. In not normally distributed data, the Mann-Whitney was used for comparison. SD was reported for the leak pressure. The statistical significance level was set at 0.05. Medcalc, version 19.6.4, was used for statistical analysis.

## Results

A total of 36 study participants were enlisted, and they were randomly assigned to one of the three study groups. Between the three groups, there were no major demographic differences (Table 1). One participant failed to attend the first training session and was thus excluded from the study. All groups improved their technical skills significantly during the training period. As a measure of technical improvement, time consumption from pretest to posttest procedures was significantly reduced in both the laparoscopic and open surgical trained groups, from 6.96 (5.04–8.88) to 2.07 (1.56–2.58) min and 7.46 (5.08–9.84) to 1.96 (1.33–2.58) min (*p* = 0.004 for both groups). The robotic surgery-trained group showed significant improvement in technical skills, increasing their MScore from 60.83 (49.73–71.94) to 87.91 (85.38–90.44) points (*p* < 0.0001).

**Table 1:** Demographics of study subjects

	Laparoscopic training group (*N* = 12)	Open surgery training group (*N* = 11)	Robotic training group (*N* = 12)
Female, *n* (%)	7 (58.3)	6 (54.5)	7 (58.3)
Age, years (range)	25 (24–26)	26 (24–27)	25 (24–27)
Played musical instrument, *n* (mean years)	5 (11.3)	0	2 (14.0)
Played organized sports, *n* (mean years)	4 (6.0)	6 (4.9)	3 (6.7)
Played video games, *n* (mean hours per week)	7 (2.9)	5 (3.5)	5 (2.1)
Prior laparoscopic simulator experience, *n* (mean total experience time (h))	3 (5.0)	1 (2.0)	1 (4.0)
Prior robotic simulator experience	0	0	0

In the final study test, one study subject interrupted before the end of the test (performed in 40 min) due to personal reasons. This study subject was evaluated on GEARS to the best of the ability of the blinded evaluators but was withdrawn from the results regarding the leak pressure test. Another study subject completed the final study test, but only 23 min of the video material was available due to technical error. This study subject was evaluated on GEARS on the present material, and the anastomosis was tested for leak pressure. Table 2 shows the distribution of study subjects completing the final study test with an end product sufficient to be tested.

**Table 2:** Distribution of study subjects completing the final study test by modality trained

	Robotic surgery	Laparoscopy	Open surgery	*p*
Completed	12	6	7	0.02
Failed completion^a^	0	6	3	

^a^Suture line that was inadequate for leak pressure test

**Fig. 2:**
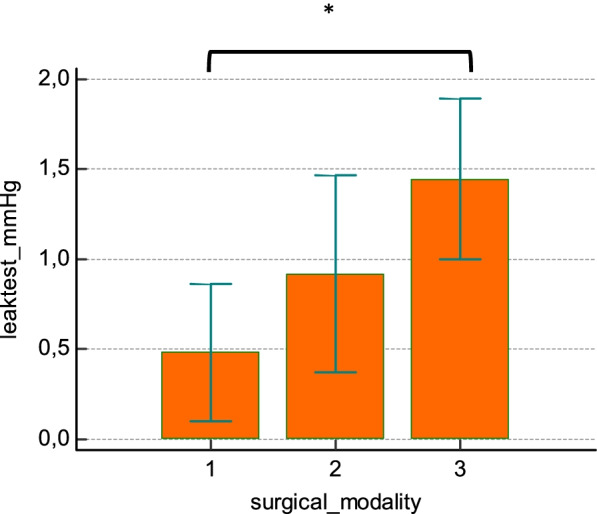
Final study test: leak pressure test on pig model robotic anastomosis. (1) Laparoscopy *n* = 12, (2) open surgery *n* = 10, and (3) robotic surgery *n* = 12. *Significant difference

The mean leak pressure of the bowel anastomosis was 36.25 (7.62–64.89) mmHg in the laparoscopic training group, 69.01 (28.02–109.99) mmHg in the open surgery group, and 108.45 (74.96–141.94) mmHg in the robotic training group. The mean anastomosis leak pressure was significantly higher in the robotic training group than in the laparoscopic training group (*p* = 0.0015), whereas there was no statistical difference when comparing the robotic training group with the open training group (Fig. 2).

**Fig. 3:**
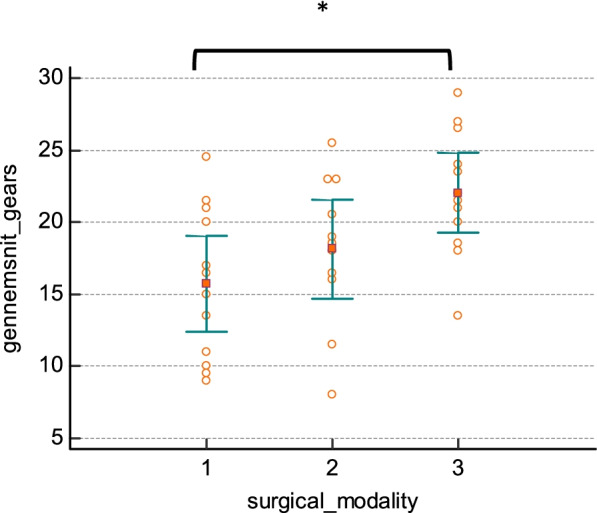
Final study test: GEARS score at test on pig model robotic anastomosis. (1) Laparoscopy *n* = 12, (2) open surgery *n* = 11, and (3) robotic surgery *n* = 12. *Significant difference

The GEARS score was 15.71 (12.37–19.04) in the laparoscopic training group, 18.14 (14.70–21.58) in the open surgery group, and 22.04 (19.29–24.79) in the robotic training group. When comparing the groups in the final study test on GEARS, the robotic training group had a statistically higher GEARS score compared to the laparoscopic training group (*p* = 0.0023). Post hoc analysis revealed no significant difference between the open and the robotic training groups (Fig. 3).

In terms of research subject characteristics, neither musical instruments nor athletic experience had a significant impact on the final test scores. When the study subjects were compared in the final study test, those who self-reported having video game experience (gamers) had a higher GEARS score than non-gamers: 21.03 4.46 versus 16.44.55 (*p* = 0.01).

## Discussion

The case volume for advanced robotic-assisted surgery is increasing exponentially, and the numbers continue to grow. However, robotic surgery requires training, and the translational aspect from other surgical modalities to robotic surgery remains elusive.

In terms of a three-dimensional surgical perspective, the use of instruments, and the degrees of freedom of movement, robotic-assisted surgery is more comparable to open surgery than laparoscopy [11]. In this study, we looked at whether open-trained surgeons would have better opportunities to transfer their surgical expertise to robotic surgery than laparoscopically trained surgeons.

We discovered that open surgery abilities transfer to robotic surgery more readily than laparoscopy does. Since we found a transfer of skills from open surgery to robotic surgery in the simulation setting, open surgery training might also serve as a foundation for the initial dexterity and economy of motion needed to accomplish basic tasks in robotic surgery.

We compared the study subjects trained in laparoscopy with the study subjects trained in open surgery regarding the transfer of skills to robotic surgery. To our knowledge, only one other study has compared the two surgical modalities in the matter of skill transfer to robotic surgery, the study by Kowalewski et al. which found that robotic-assisted surgery requires skills distinct from both conventional laparoscopy and open surgery [1]. Kowalewski et al. had a heterogeneous study group with different ages and levels of surgical experience. The strength of our study is the homogenous group constellation as we included exclusively novice study subjects. Because the study logistics constraints limited the number of participants to 36, we did not do a power calculation. It would be relevant to make a study with larger sample size and residents in a surgical field instead of medical students with no prior surgical experience. Medical students do not accurately portray surgical residents, who are all familiar with tissue management and have a variety of surgical experiences. By investigating a more advanced component of the robotic learning curve, we should be able to acquire a better understanding of the impact of prior surgical experience on robotic skills. However, utilizing surgical residents would increase variability.

In the study’s training phase, we used fresh tissue models to mimic a clinical environment. We modified a simulation model previously used as a training tool for laparoscopic [16, 17], robotic [16], and open surgical [13, 18] simulations. The simulated robotic surgical tasks in the final test were performed as a higher fidelity simulation with a live pig model, incorporating components like tissue manipulation, dissection, and anatomical understanding. This was done to match the clinical setting as closely as possible. The bowel anastomosis exercise is a well-recognized test for assessing technical surgical skills [19, 20]. We employed it as a technique of evaluation for technical surgical abilities well aware of its limitations in terms of addressing all relevant skills in surgery, and thereby, performance was assessed using both previously verified subjective metrics and objective measures (GEARS). Furthermore, professional robotic surgeons blinded to the study subjects’ identities and training modality assessed the performance in the final evaluation.

The study was designed having a fixed time in training sessions instead of proficiency-based training. This could be considered a limitation of the study; however, flexibility in time for individual improvement of skills would not be feasible, due to study logistics. Finally, it was the same experienced instructor for all training sessions. The choice of a single instructor can also be regarded as a limitation because it might introduce bias into the study’s training component. However, due to the fact that the same procedure was done repeatedly with a clear end-product, the trainer’s role was quickly reduced to a passive one, since we believe it is less important for the outcome.

The importance of open surgical training in creating a robotic curriculum appears to be underrated. Future studies could determine the proficiency level of open surgery skills advantageous to achieve prior to robotic training. Robotic-assisted training is expensive and time-consuming. Access to a robotic system and robotic training tools is required for robotic simulation training, which is costly and not necessarily accessible at training institutes. Robotic virtual reality (VR) training requires access to expensive simulators. By potentially shortening the learning curve for robotic surgery training by achieving basic skills on low-fidelity easy-accessible open surgery simulation, time and money could be saved.

## Conclusion

In our study, training in open surgery is superior to training in laparoscopy when transitioning to robotic surgery in a simulation setting performed by surgically naive study subjects. Given the extensive use of robotic surgery and the limited time available for training, these findings may aid in the construction of a robotic surgery training curriculum.

## Data Availability

The data generated and analyzed during the current study are available from the corresponding author upon request.

## Abbreviations

GEARS: Global Evaluative Assessment of Robotic Skills
dVSS: da Vinci Skills Simulator
VR: Virtual reality
